# Pre-Exercise Nutrition: The Role of Macronutrients, Modified Starches and Supplements on Metabolism and Endurance Performance

**DOI:** 10.3390/nu6051782

**Published:** 2014-04-29

**Authors:** Michael J. Ormsbee, Christopher W. Bach, Daniel A. Baur

**Affiliations:** 1 Human Performance and Sports Nutrition Lab, Department of Nutrition, Food, and Exercise Sciences, Florida State University, Tallahassee, FL 32306, USA; E-Mails: cwb12b@my.fsu.edu (C.W.B.); dab13b@my.fsu.edu (D.A.B.); 2 Biokinetics, Exercise and Leisure Sciences, University of KwaZulu-Natal, Durban, 4000, South Africa

**Keywords:** carbohydrate, fat, protein, performance, caffeine, beet root juice, dietary nitrate, glycemic index, nutrient timing

## Abstract

Endurance athletes rarely compete in the fasted state, as this may compromise fuel stores. Thus, the timing and composition of the pre-exercise meal is a significant consideration for optimizing metabolism and subsequent endurance performance. Carbohydrate feedings prior to endurance exercise are common and have generally been shown to enhance performance, despite increasing insulin levels and reducing fat oxidation. These metabolic effects may be attenuated by consuming low glycemic index carbohydrates and/or modified starches before exercise. High fat meals seem to have beneficial metabolic effects (e.g., increasing fat oxidation and possibly sparing muscle glycogen). However, these effects do not necessarily translate into enhanced performance. Relatively little research has examined the effects of a pre-exercise high protein meal on subsequent performance, but there is some evidence to suggest enhanced pre-exercise glycogen synthesis and benefits to metabolism during exercise. Finally, various supplements (*i.e.*, caffeine and beetroot juice) also warrant possible inclusion into pre-race nutrition for endurance athletes. Ultimately, further research is needed to optimize pre-exercise nutritional strategies for endurance performance.

## 1. Introduction

Optimal endurance performance requires careful consideration of nutrient intake. Research accumulated over the last half-century has shown that the most beneficial nutritional intervention is one that can augment and preserve carbohydrate (CHO) fuel stores (muscle and liver glycogen) for late-race, high-intensity exercise. Consuming a meal in the hours preceding an event is one method for maximizing glycogen stores and potentially influencing its utilization during exercise. The aim of the following review is to examine the effects of pre-exercise macronutrient composition on metabolism and performance. Additionally, the metabolic and performance effects of consuming caffeine and beetroot juice in the hours prior to exercise will be discussed.

## 2. Carbohydrate-Rich Meals

Pre-exercise CHO ingestion has been a topic of controversy in recent years [1], likely stemming from its well-known metabolic effects. Consumption of CHO leads to a substantial increase in plasma glucose [2]. As a result, insulin is released from the pancreas [3], and hepatic glucose output is blunted [4,5]. Insulin initiates a signaling pathway in muscle, resulting in GLUT4 translocation and glucose uptake into the muscle cell [5]. Increased glucose availability within muscle stimulates glycolysis and glucose oxidation [6,7,8]. Simultaneously, insulin reduces fat oxidation [2]. This shift in substrate utilization seems to be primarily explained by insulin-mediated inhibition of lipolysis, which reduces free fatty acid (FFA) availability [9]. However, increased glucose uptake and oxidation reduces fat oxidation even in the presence of high intracellular concentrations of FFA [7], possibly as a result of reduced FFA transport into the mitochondria [10].

When CHO ingestion precedes exercise (up to 6 h prior [11]), hyperinsulinemia, in combination with enhanced exercise-induced GLUT4 translocation [12], serves to reduce blood glucose concentrations, potentially causing early-exercise hypoglycemia in some individuals [2,6]. Additionally, some, but not all [13,14], data indicate increased glycogenolysis, likely as a result of insulin-modulated FFA oxidation during exercise [6,15,16]. These combined effects suggest a resultant reduced CHO availability late in exercise that could potentiate the early onset of fatigue. However, most studies show either no negative effects or enhanced performance with pre-exercise CHO ingestion (discussed below).

### 2.1. Carbohydrate Feedings and Performance

In general, ingesting CHO prior to exercise appears to be beneficial to performance (see Table 1). Of all the studies to examine the topic, only one early study by Foster *et al.* [17] reported reduced time to exhaustion (TTE) when pre-exercise CHO ingestion was compared to a placebo. Conversely, subsequent research has reported either no functional differences (between CHO and placebo) [14,18,19,20,21,22,23,24,25] or enhanced exercise capacity [26,27,28,29,30,31,32,33] and time trial (TT) performance [28,34] with pre-exercise CHO intake.

**Table 1:** The effect of pre-exercise carbohydrate feedings on performance or exercise capacity.

Study	Year	*n*	Treatments	Timing Prior	Protocol	Results	Performance
Foster *et al.* [17]	1979	16	P, 75 g G, mixed beverage	30 min	TTE cycling 100% and 80% VO_2max_	43.2 min (G) *vs.* 53.2 min (P)	↓
McMurray *et al.* [26]	1983	6	P, 90 g G, 90 g F	45 min	TTE running 80% VO_2max_	63.9 min (G) *vs.* 52.2 min (P)	↑
						61.9 min (F) *vs.* 52.2 min (P)	↑
Devlin *et al.* [18]	1986	8	P, CHO bar (43 g, 9 g fat, and 3 g protein)	30 min	TTE intermittent cycling 70% VO_2max_ (15 min exercise, 5 min rest)	52 min (CHO) *vs.* 48 min (P)	↔
Hargreaves *et al.* [14]	1987	6	P, 75 g G, 75 g F	45 min	TTE cycling 75% VO_2max_	92.8 min (G) *vs.* 92.7 min (P)	↔
						90.6 (F) *vs.* 92.7 (P)	↔
Okano *et al.* [30]	1988	12	P, 60 g (F), 85 g (F)	60 min	TTE cycling ~80% VO_2max_ (following 65% VO_2max_ preload)	145 min (F) *vs.* 131 min (P)	↑
Sherman *et al.* [41]	1991	9	P, 1.1 g/kg BM G (LC), 2.2 g/kg BM G + MD (HC)	60 min	90 min cycling 70% VO_2max_, ~45 min TT	LC and HC avg. 12.5% faster *vs.* P	↑
Thomas *et al.* [27]	1991	8	P, 1 g/kg BM CHO-lentils (LGI), potato (HGI), G	60 min	TTE cycling 70% VO_2max_	LGI *vs.* P	↑
						HGI *vs.* P	↑
						G *vs.* P	↑
						LGI *vs.* HGI	↑
Wright *et al.* [28]	1991	9	P, 5 g/kg BM (G + Suc)	3 h	TTE cycling 70% VO_2max_	237 min (G + Suc) *vs.* 201 min (P)	↑
Chryssanthopoulos *et al.* [19]	1994	9	P, 75 g G	30 min	TTE running 70% VO_2max_	133.8 min (G) *vs.* 121.2 (P)	↔
Sparks *et al.* [20]	1998	8	P, 1 g/kg BM (HGI), 1 g/kg BM (LGI)	45 min	50 min cycling at ~65% VO_2max_, 15 min TT	249 kJ (HGI) *vs.* 254 kJ (P)	↔
						253 kJ (LGI) *vs.* 254 kJ (P)	↔
Whitley *et al.* [21]	1998	8	P, HCM (215 g CHO, 26 g protein, 3 g fat), HFM (50 g CHO, 14 g protein, 80 g fat)	4 h	90 min cycling 70% VO_2max_, 10 km TT	14.63 min (HCM) *vs.* 14.56 min (P)	↔
						14.23 min (HFM) *vs.* 14.56 min (P)	↔
Schabort *et al.* [31]	1999	7	Fasted, 100 g CHO	3 h	TTE cycling 70% VO_2max_	136 min (CHO) *vs.* 109 min (fasted)	↑
Febbraio *et al.* [22]	2000	7	P, 2 g/kg BM G	30 min	120 min cycling 70% VO_2max_, ~45 min TT	G finishing time no different *vs.* P	↔
Febbraio *et al.* [8]	2000	8	P, 1 g/kg BM (HGI), 1 g/kg BM (LGI)	30 min	120 min cycling 70% VO_2max_, 30 min TT	LGI not different from P	↔
						HGI not different from P	↔
						HGI not different from LGI	↔
Kirwan *et al.* [29]	2001	6	P, 75 g (MGI), 75 g (HGI)	45 min	TTE cycling 60% VO_2max_	165 min (MGI) *vs.* 141 min (P)	↑
						134 min (HGI) *vs.* 141 min (P)	↔
Chryssanthopoulos *et al.* [32]	2002	10	P+P, HCM (2.5 g/kg BM CHO) + P, HCM + G (6.9% CHO drink)	3 h	TTE running 70% VO_2max_	112 min (HCM+P) *vs.* 103 (P+P)	↑
						125 min (HCM+G) *vs.* 103 (P+P)	↑
Jentjens *et al.* [24]	2003	9	P, 25 g G, 75 g G, 200 g G	45 min	20 min cycling 65% VO_2max_, ~40 min TT	43.3 min (25g G) *vs.* 42.5 min (P)	↔
						43.1 min (75g G) *vs.* 42.5 min (P)	↔
						42.2 min (200g G) *vs.* 42.5 min (P)	↔
Pritchett *et al.* [25]	2008	10	P, bar (20 g CHO, 12 g protein and 4.5 g fat)	15 min (S), 60 min (L)	Repeated cycling Wingate bouts	112 kJ (S) *vs.* 106 kJ (P)	↔
						115 kJ (L) *vs.* 106 kJ (P)	↔
Tokmakidis *et al.* [33]	2008	11	P, 1 g/kg BM G	15 min	TTE running 80% VO_2max_ (following 5 min 60% VO_2max_ and 45 min 70% VO_2max_)	83 min (G) *vs.* 76 min (P)	↑
Chen *et al.* [34]	2009	8	P, ~100 g CHO (LGI), ~100 g CHO (HGI)	2 h	5 km running 70% VO_2max_, 16 km TT	91.5 min (HGI) *vs.* 93.6 min (P)	↑
						92.4 min (LGI) *vs.* 93.6 min (P)	↔
						92.4 min (LGI) *vs.* 91.5 min (HGI)	↔

Notes: P, placebo; G, glucose; TTE, time to exhaustion; F, fructose; CHO, carbohydrate; BM, body mass; LC, low carbohydrate; MD, maltodextrin; HC, high carbohydrate; TT, time trial; VO_2max_, maximal oxygen consumption; HCM, high carbohydrate meal; HFM, high fat meal; kJ, kilojoule; LGI, low glycemic index; HGI, high glycemic index; Suc, sucrose; km, kilometer; MGI, moderate glycemic index.

Any performance benefit derived from consuming CHO prior to exercise is likely a result of increased glycogen storage [2]. During an overnight fast, liver glycogen stores are reduced substantially, with some studies reporting glycogenolysis rates of ~0.2–0.3 mmol glucosyl units per min during the fast [35,36], which equates to an approximate 80% reduction in liver glycogen stores overnight [37]. Thus, sub-optimal CHO stores are likely to be present when beginning to exercise in the fasted state. Consumption of CHO prior to exercise can maximize glycogen storage. Indeed, with the use of nuclear magnetic resonance (NMR) spectroscopy, Taylor *et al.* (1996) reported that following the consumption of a mixed meal, ~20% of ingested CHO is directly stored as liver glycogen [38]. Moreover, Coyle and colleagues [2] reported a 42% increase in muscle glycogen storage following pre-exercise ingestion of CHO. Therefore, consuming CHO may help to increase CHO availability by maximizing CHO fuel stores prior to exercise. As such, benefits to performance may be more apparent in long-duration (>2 h) performance trials, which are likely limited by initial glycogen content [39,40]. Indeed, many studies report pre-exercise CHO-mediated enhanced performance in long-duration TTE trials or pre-loaded TT [28,29,30,31,32,41]. Alternatively, in studies utilizing performance trials <2 h, many studies report no change [14,18,20,21,24,25], and relatively few report enhanced performance [26,33,34]. Worth noting, enhanced performance in shorter trials could be explained by non-metabolic, centrally-mediated effects on motor output stemming from the stimulation of CHO receptors in the mouth [42].

### 2.2. Effects of Timing

The timing of CHO intake influences its metabolic effects. Indeed, insulin and blood glucose elevations are positively correlated with CHO meal proximity to exercise [43]. Studies in which CHO is consumed 1–4 h prior to exercise often report glucose and insulin levels declining to near-basal levels prior to exercise [2,32,34]. Alternatively, when subjects consume CHO ≤60 min before exercise, insulin and blood glucose levels are reported to be elevated immediately prior to exercise [4,19,23,44]. Interestingly, regardless of the timing of ingestion and the degree of blood glucose or insulin elevation prior to exercise, the metabolic perturbations associated with CHO meal ingestion result in an initial drop in blood glucose at the start of exercise [2,4]. Although this initial drop is transient and blood glucose levels typically increase to basal levels within ~20 min [4,20,43,45], it is worth noting that the degree to which blood glucose is reduced early in exercise seems to be associated with meal proximity, with meals ingested ≤60 min pre-exercise resulting in a greater reduction [43].

The effect of the timing of pre-exercise CHO ingestion on performance is less clear. Studies investigating the consumption of CHO at various time points within 75 min of exercise have reported no influence of timing on performance [25,43]. However, no studies have compared the effects of ingesting CHO within the hour before exercise *versus* 2–3 h prior to exercise. Of interest, in studies where subjects consumed a CHO meal 2–3 h prior to exercise, TTE [28,31,32] and TT performance [34] were consistently improved. Alternatively, studies comparing CHO ingestion to placebo ≤60 min prior to exercise have reported far less consistent effects on performance (see Table 1). With this in mind, it seems reasonable that the metabolic perturbations associated with consuming CHO within 1 h prior to exercise (*i.e.*, a large increase in blood glucose and insulin followed by a dramatic drop in blood glucose [43]), although seemingly inconsequential when compared to placebo, may potentially impair performance when compared to CHO ingestion 2–3 h prior. Thus, it may be prudent for those susceptible to hypoglycemia to schedule their pre-race meal 2–3 h prior. Alternatively, one might consider consuming CHO during warm-up, as this has been reported to blunt pre-race glucose and insulin spikes, likely as a result of catecholamine-induced insulin inhibition [46].

### 2.3. Effects of Glycemic Index

The glycemic index (GI) refers to the degree to which a CHO elevates blood glucose in the 2 h following consumption [47]. The higher the GI of a CHO, the more rapid is the increase in blood glucose. As such, consuming a low- *versus* high-GI CHO prior to exercise results in an attenuated blood glucose and/or insulin response [20,23,48,49,50,51,52], which may help to enhance fat oxidation and/or maintain euglycemia during exercise [20,29,48,49,53,54,55], although not all studies support this [56,57]. This potentially enhanced fat oxidation may be the reason why some have noted a trend for muscle glycogen sparing with low- *versus* high-GI CHO [23]. Alternatively, high-GI CHO, as noted above, may increase glycogenolysis [6,15,16]. With this in mind, it seems logical that the beneficial metabolic effects from consuming a low-GI CHO would benefit performance *versus* high-GI CHO via preservation of endogenous glycogen stores. However, findings have been inconsistent, with some reporting enhanced TTE [29,50,54] and TT performance [49,58] with low-GI CHO and others reporting no differences *versus* high GI CHO [20,23,48,51,52,55]. These inconsistencies may be due to methodological differences (e.g., timing, amount of CHO, exercise protocol). Of note, no studies report a performance decrement resulting from low-GI CHO consumption *versus* high-GI CHO. As such, low-GI CHO represents an intriguing pre-exercise nutritional option that may benefit performance to a greater extent or, at least, equally to high-GI CHO. More research is warranted to determine the true effects of low-GI CHO, as the confirmation of its potential superiority over high-GI CHO would be quite relevant to athletes.

### 2.4. Multiple Transportable Carbohydrates

Besides the glycemic effects of pre-exercise CHO, other important considerations mediated by CHO type include gastric emptying, fluid delivery, absorption rates and the effects on gastrointestinal comfort. Generally, gastric emptying and fluid delivery are negatively correlated with the energy content of CHO being ingested [59]. Absorption is a function of both gastric emptying and intestinal transporter number and activity [60]. Gastrointestinal distress may result from malabsorption of ingested CHO [61]. All of these variables can significantly influence the rate of CHO availability for glycogen synthesis prior to exercising, as well as the incidence of gastrointestinal distress during exercise itself.

Recent research indicates that the type and/or composition of CHO can influence gastric emptying, fluid delivery, absorption and gastrointestinal distress. For example, studies examining the effects of consuming glucose/maltodextrin + fructose (GF) during exercise have reported enhanced gastric emptying, fluid delivery and absorption rates *versus* isocaloric amounts of glucose alone [62,63]. Moreover, consuming GF during exercise seems to attenuate gastrointestinal distress when compared to isocaloric glucose alone [64,65]. These effects may be due to non-competitive CHO intestinal transport, as glucose and maltodextrin are transported into the blood stream via the sodium-dependent SGLT1 transporter and fructose via GLUT5 [66]. This “multiple transport” of CHO seems to enhance the delivery of CHO to muscle. Indeed, research has indicated that the oxidation of exogenous CHO during exercise is significantly higher with GF (1.75 g/min) *versus* isocaloric glucose alone (~1.0 g/min) [63]. This may explain the reported performance improvements with during-exercise GF *versus* isocaloric glucose alone [64,65,67,68].

While the ingestion of multiple transportable CHO (MTC) during exercise has been thoroughly researched, the effects of MTC ingestion pre-exercise have not been investigated. Presumably, due to enhanced gastric emptying, fluid delivery and CHO absorption, MTC could provide a more effective means of hydrating and increasing CHO availability prior to exercise. Furthermore, although purely speculative, if an athlete is limited to or prefers to consume additional CHO ≤1 h prior to exercising, the enhanced digestion of MTC *versus* glucose alone may help to improve gut comfort during exercise. Clearly, future research on the effects of pre-exercise MTC is warranted.

### 2.5. Modified and Resistant Starches

Interestingly, technological advances have allowed for the modification of starches via various means (e.g., hydrothermally modified starch, acid/alcohol-modified cornstarches, chemical modified starches) and represents a novel method to potentially enhance CHO availability during exercise [69,70,71]. As discussed above, the importance of CHO availability to exercise performance is well-established [72], and it can be accomplished in one of three ways: (1) providing exogenous CHO to serve as a fuel source; (2) altering substrate utilization in a way that preserves endogenous CHO; or (3) a combination of the two. Traditionally, starches can be classified as either slowly or rapidly digestible based on their rate of glucose release and their absorption rate upon ingestion [73]. More specifically, the varying rates of digestibility are traditionally dependent upon the amylose:amylopectin ratio of their structural makeup, with a higher ratio increasing the resistance to digestion (for a thorough review of starch structure and digestibility, see [74]). However, by modifying starches, the gastric-emptying rate can be manipulated despite the amylose:amylopectin ratio. Thus, these modification techniques can either enhance or inhibit the glycemic and insulinemic responses. Logically, this could enhance glycogen storage pre- or post-exercise or spare glycogen during exercise by enhancing fat oxidation.

While there is some evidence of beneficial metabolic effects from consuming modified starches in certain clinical populations (e.g., diabetics) [75,76,77,78,79,80,81], data on the performance effects is limited [69,71,82,83,84] (Table 2). Moreover, because the type of modification results in either a slow- or fast-digesting starch, it is important to view the limited data based upon the digestion rate. As such, fast-digesting modified starches, such as Vitargo^®^, may benefit performance. Stephens *et al.* [84] examined the implications of consuming a high molecular weight (HMW) rapidly-digested modified starch (Vitargo^®^), a low molecular weight (LMW) glucose polymer derived from hydrolyzed corn starch representing a maltodextrin recovery drink or sugar-free water on muscle glycogen resynthesis and endurance performance. Following a glycogen-depleting, submaximal cycling test (75% of maximal oxygen uptake (VO_2max_), subjects consumed one of the aforementioned drinks and then rested for 2 h. Immediately after the 2-h rest, those who consumed the HMW or LMW starch exhibited a significantly greater work output on a 15-min all-out cycling test in comparison to the sugar-free water group (*p* < 0.001 and *p* < 0.01, respectively). In addition, the HMW group had a 10% increase in work output in comparison to the LMW group (*p* < 0.01) [84]. Thus, this fast-digesting modified starch seems beneficial when consumed between exercise bouts; however, its effects on performance when consumed <2 h pre-exercise has not been investigated.

**Table 2:** The effect of pre-exercise or refeeding of modified and resistant starches on endurance performance.

Study	*n*	Starch Type	Treatments	Timing Prior	Protocol	Results	Performance
**Slow-Digesting**							
Roberts *et al.* [83]	9	Waxy (95% amylopectin), hydrothermally modified	1 g/kg BM MS, 1 g/kg MD	30 min	150 min cycling 70% VO_2max_, TTE 100% VO_2max_	125 s (MS) *vs.* 136 s (MD)	↔
Goodpaster *et al.* [82]	10	Waxy (100% amylopectin) or resistant (100% amylose)	1 g/kg BM WMS, RMS, G or P	30 min	90 min cycling 66% VO_2max_, 30 min TT	434 kJ (G) *vs.* 403 kJ (P)	↑
						428 kJ (WMS) *vs.* 403kJ (P)	↑
						418 kJ (RMS) *vs.* 403 kJ (P)	↔
						G *vs.* WMS *vs.* RMS	↔
Jozsi *et al.* [69]	8	Waxy (100% amylopectin) or resistant (100% amylose)	3,000 kcal (65:20:15% carbohydrate, fat and protein); CHO consisting‚ of WMS, RMS, G or MD	Post-exercise consumption over 12 h	60 min cycling 75% VO_2max_, 6 × 1 min at 125% VO_2max_, 24 h rest, 30 min TT	422 kJ (WMS), 413 kJ (RMS), 431 kJ (G), and 423 kJ (MD)	↔
**Fast-Digesting**							
Stephens *et al.* [84]	8	Low molecular weight and high molecular weight glucose polymers	100 g LMS or HMS, P	Post-exercise feeding 2 h prior to second bout	TTE cycling 73% VO_2max_, 2 h rest, 15 min TT	149 kJ (LMS) *vs.* 138 kJ (P)	↑
						164 kJ (HMS) *vs.* 138 kJ (P)	↑
						164 kJ (HMS *vs.* 149 kJ (LMS)	↑

Notes: BM, body mass; MS, modified starch; MD, maltodextrin; TTE, time to exhaustion; WMS, waxy modified starch; RMS, resistant modified starch; G, glucose; P, placebo; TT, time trial; kJ, kilojoule; LMS, low molecular weight modified starch; HMS, high molecular weight modified starch; VO_2max_, maximal oxygen consumption.

In contrast, Roberts *et al.* [83] examined the effects of an HMW slow-digesting hydrothermally modified starch (UCAN^®^) in comparison to maltodextrin on metabolic responses to a 150-min submaximal cycling bout (70% VO_2peak_) followed by a TTE (100% VO_2peak_). While the authors report blunted glycemic and insulin responses and increased fat oxidation as a result of HMW slow-digesting starch consumption in comparison to maltodextrin, no significant difference was observed in TTE performance at 100% VO_2peak_ (HMS (high molecular weight modified starch): 125 ± 28 sec *vs.* maltodextrin: 136 ± 27 sec; *p* = 0.66).

Overall, modifying starches appears to enhance CHO availability in multiple ways. Despite the Stephens *et al.* [84] and Roberts *et al.* [85] studies both using HMW starches, the different modification techniques yielded starches with varying metabolic and performance outcomes. The fast-digesting Vitargo^®^ shows an ability to rapidly raise glucose and insulin levels. Although not directly measured, these metabolic effects likely aid in the resynthesis of glycogen stores following exercise, which may explain the enhanced performance in subsequent exercise [84]. In contrast, slow digesting modified starches offer the potential for a blunted glucose and insulin response, aiding in the increased utilization of FFAs during exercise; however, there appears to be no effect on performance *versus* simple CHO (e.g., glucose or maltodextrin) [69,82,83] (Table 2).

## 3. High-Fat Meals

With the importance of endogenous CHO stores on endurance performance well established, recent studies have begun to examine various nutritional and training methods with the aim of optimizing performance through the manipulation of substrate utilization during exercise. The prevailing concept behind the majority of this research is to use macronutrient manipulation to determine the correct interplay between maximizing endogenous CHO storage and optimizing the capacity for fat oxidation to ultimately improve endurance performance. Due to the fact that endurance training has been shown to increase the metabolic capacity to oxidize fat during submaximal exercise [86], it seems logical that increasing the ability of endurance athletes to utilize an alternative fuel source to CHO (*i.e.*, fat) would improve endurance performance.

Consumption of a high-fat meal pre-exercise alters substrate supply before exercise and leads to increased free fatty acid (FFA) levels in the blood [21]. Increased FFA levels will increase lipid metabolism during exercise [87,88,89,90] and either preserve endogenous CHO stores [6,91,92,93] or attenuate the normal rate of CHO depletion [87,88,90,91,92,94,95]. While studies have shown significant performance enhancement as a result of increasing fat availability (via diet) in animals [96,97,98] or in humans with heparin administration [6,91,92,93], the effects of consuming a high fat meal on subsequent exercise performance are equivocal.

### 3.1. Fat Adaptation and Performance

While the focus of this review is centered on acute pre-exercise feedings, it is important to note that the effect of fat adaptation has been examined in both short-term (1–4 days) [39,99,100,101,102,103] and long-term (7–28 days) scenarios [87,88,90,104,105,106,107,108,109]. Various studies have identified metabolic adaptations resulting from these types of diets, such as increased fat oxidation compared to high-CHO diets [86,87,88,90,109,110] and the sparing of endogenous CHO stores [91,92,93]. However, little evidence indicates that fat adaptation (as a result of high-fat, low-CHO diets) whether short term or long term, improves endurance performance [90]. While speculative, it is plausible that this feeding strategy may ultimately show benefits for moderate-intensity, ultra-distance endurance performance, which favors fat as the primary fuel source [111] (for a more thorough review of the effect of short- and long-term fat adaptation on performance, see [112,113]).

### 3.2. Acute High-Fat Ingestion and Performance

In contrast to a “fat adaptation” approach over a period of days or weeks, another method to improve performance is to increase fat availability acutely through the consumption of a high-fat meal within the hours (≤4 h) prior to exercise. While chronic (≥1 week) consumption of a high-fat, low-CHO diet impairs endurance performance as a result of decreasing endogenous CHO stores [39,99,100,102,103], consuming a single high-fat meal prior to exercise theoretically would allow for both maximal endogenous CHO storage as a result of traditional CHO-loading in the days prior to the event [114], as well as immediate fatty acid availability from the pre-exercise meal [21,115,116]. However, despite these metabolic benefits, most studies report no performance benefits from consuming a pre-exercise high-fat meal when compared to a high-CHO meal [21,89,115,117].

Interestingly, Murakami *et al.* [116] recently examined the performance effect of consuming either: (1) a high-fat meal 4 h pre-exercise + a placebo jelly 3 min before exercise (HFM + P); (2) a high-fat meal 4 h pre-exercise + maltodextrin jelly 3 min before exercise (HFM + M); or (3) a high-CHO meal 4 h pre-exercise + placebo jelly 3 min before exercise (HCM + P); after consuming an isocaloric, high-CHO diet for three days (2562 ± 19 kcal). Meals consumed 4 h pre-exercise were isocaloric (1007 ± 21 kcal); however, maltodextrin added 410 ± 8 kcal, while the placebo added 0 kcal. This double-blind, crossover study [116] tested eight collegiate male distance runners (mean VO_2max_ of 61.3 ± 2.2 mL/kg/min) for an 80 min submaximal run on a treadmill at each runner’s pre-determined lactate threshold (LT) speed, immediately followed by a time trial to exhaustion (TTE). Participants in the HFM + M group exhibited both a significantly higher fat oxidation rate and a significantly decreased CHO oxidation rate during the first 60 min of exercise compared to the HCM + P group. This suggests that CHO feeding subsequent to a HFM pre-exercise and three days of a proper CHO loading protocol can elicit an enhancement in the endurance performance of well-trained runners. The increased fat oxidation and decreased CHO oxidation during the first 60 min of exercise theoretically leads to an increase in glycogen stores at the end of exercise, thus improving TTE performance. Worth noting, a significant increase in TTE duration in the HFM + M group (100 ± 3.4 min) compared to the HFM + P (92 ± 2.8 min) or HCM + P groups (90 ± 1.7 min) was reported; however, Murakami and colleagues did not include a HCM + M group, which raises questions about whether the HFM + M group performed longer primarily due to HFM or rather as a result of the increased caloric consumption of maltodextrin immediately pre-exercise. Furthermore, these findings are in direct contrast to others comparing pre-exercise high-CHO meal consumption to high fat and should be interpreted with caution, due to methodological considerations [21,89,115,117]. Thus, further study is warranted.

Worth noting, another potential factor that may need to be addressed is the fact that the majority of research methodology pertaining to acute pre-exercise fat feeding has used cycling as the exercise modality [21,89,115,117]. The work of Murakami *et al.* [116] prompts the questioning of the importance of the exercise modality in eliciting significant changes. Though strictly theoretical, perhaps the muscle recruitment mechanics of the individual exercise modalities differentially influences the metabolic effects resulting from various pre-exercise macronutrient manipulations.

## 4. Mixed CHO-Protein Meals

Much research has been done in recent years investigating the effects of adding protein (PRO) to CHO (CHO-PRO) beverages or supplements during exercise and post-exercise. Findings have been intriguing with some, but not others [118,119], reporting enhanced TTE [120,121,122] and TT performance [123] with during-exercise CHO-PRO *versus* CHO intake alone. Additionally, some, but not all [124,125,126,127,128,129,130], studies investigating the effects of post-exercise CHO-PRO intake on subsequent exercise performance have also noted enhanced TTE [131,132] and TT performance [133], possibly as a result of increased glycogen resynthesis [127,134,135,136]. Despite these findings, there has been very little research done analyzing the effects of pre-exercise CHO-PRO in the performance context. Thus, a complete understanding of CHO-PRO pre-exercise effects requires the examination of research in non-athletic populations.

Research examining the clinical implications of CHO-PRO consumption prior to exercise has helped to elucidate its metabolic effects. Of interest, adding PRO to CHO seems to attenuate the glycemic response compared to CHO alone [85,137,138]. These effects may be partly explained by PRO-induced hormonal alterations, which are attributed to elevated levels of certain amino acids in the blood [138,139]. Specifically, elevations in arginine, leucine and phenylalanine stimulate both β and α cells of the pancreas, resulting in the secretion of both insulin and glucagon, respectively [140]. While the PRO-induced glucagon reaction is completely unique from CHO intake, the post-feeding insulin rise is also distinctively high with CHO-PRO *versus* CHO, because insulin seems to respond additively to glucose and amino acid elevations [85,141,142]. The combined effects of these hormonal increases may, via insulin, enhance glucose disposal [141] and, simultaneously, via glucagon, stimulate hepatic glucose output [143], thereby helping to maintain euglycemia. It is also worth noting that due to higher insulin levels with CHO-PRO, FFA oxidation may be reduced to a greater degree *versus* CHO [9,144]. However, this effect may be partially counterbalanced by the potentially lipolytic effects of glucagon [145,146].

Besides the potential protection from early-exercise hypoglycemia, CHO-PRO ingestion may also enhance pre-exercise fuel storage. Several mechanistic studies in rodents have determined that pre-exercise PRO consumption can enhance glycogen synthesis [147,148,149] and may lead to glycogen sparing during exercise [149]. With this evidence in rodents in combination with the evidence of enhanced post-exercise glycogen resynthesis [127,134,135,136], it seems plausible that pre-exercise CHO-PRO ingestion could augment glycogen storage pre-exercise in humans. However, while it is tempting to speculate that CHO-PRO ingestion prior to exercise could enhance exercise capacity or performance in humans by augmenting and/or sparing glycogen stores, there is little evidence to support or refute this notion [115].

### 4.1. Protein Feedings and Performance

To our knowledge, there is only one study [115] analyzing the effects of a PRO meal on subsequent endurance exercise metabolism and performance. Using trained cyclists, Rowlands and Hopkins [115] investigated the effects of the pre-exercise (90 min) ingestion of three different fuels ((1) CHO; (2) PRO; or (3) a high-fat meal) on late-exercise TT and sprint performance (following ~2 h of cycling). Although the authors reported no differences in performance, metabolic differences were apparent. Specifically, the CHO meal increased insulin and decreased FFA oxidation levels to a greater degree than fat or PRO. These findings are somewhat unexpected based on reports of higher insulin levels [85,141,142] and lower FFA oxidation with CHO-PRO intake *versus* CHO alone [144]. However, in this study, soy protein was utilized, which may have influenced the insulinemic response differently from other types of protein (e.g., whey) [150,151]. Perhaps the use of whey PRO would have resulted in a greater insulin response, potentially enhancing glycogen storage and during-exercise metabolism, although this idea is purely speculative. Further worth noting, subjects in this study consumed a CHO drink during exercise. The maintenance of plasma glucose levels in all trials may have blunted the effects of a PRO meal, which may explain why no performance differences were observed. Furthermore, the long trial duration (3+ h) may have influenced reliability (3.7% within-subject error for 50 km TT), making it more difficult to detect statistical differences. Therefore, more research is necessary to determine the true effects of pre-exercise CHO-PRO consumption.

## 5. Supplements and Other Considerations

### 5.1. Caffeine

Caffeine use to improve endurance performance has been extensively studied and reported elsewhere (see [152,153,154] for detailed reviews). Therefore, this section is not intended to be a comprehensive review, but rather, a brief overview focused on recent publications pertaining to caffeine use pre-exercise for endurance performance.

Recent research on the effects of pre-exercise caffeine consumption has generally supported it as an ergogenic aid. Indeed, in line with the original work by Costill and colleagues [155], many studies have reported enhanced TT [156,157,158,159,160,161], TTE [162,163] and total work completed in a given time [164,165] with pre-exercise caffeine consumption; however, not all studies agree [166]. These beneficial performance effects seem to be both repeatable [159] and unaffected by caffeine habituation [162,167]. Moreover, the ergogenic effects may be more apparent in trained individuals rather than healthy, untrained individuals [158]. With this in mind, this finding may also be related to recent findings from Womack *et al.* [168], suggesting that the degree of performance enhancement derived from pre-exercise caffeine consumption may be strongly influenced by genetics. In this study, using a randomized cross-over design, 35 trained male cyclists received a placebo or 6 mg/kg of caffeine prior to a simulated 40-km TT [168]. The authors reported that caffeine enhanced performance to a greater degree in those with a specific polymorphism (homozygous for the A allele) of the CYP1A2 gene [168]. This evidence suggests that there may be responders and non-responders to caffeine. Regardless of an athlete’s genetic disposition, a dose of 3–6 mg of caffeine/kg of body weight has been shown to enhance performance in most individuals [152], with no further benefit from higher doses [169].

Multiple mechanisms of action for caffeine’s performance effects have been proposed [170] that include: (1) blocking adenosine receptors [152,171]; (2) increasing lipolysis and sparing glycogen [172]; (3) inhibiting phosphodiesterase activity, allowing cyclic adenosine monophosphate (cAMP) to stay active longer [173]; (4) increasing glycogen resynthesis after exercise [174]; and (5) enhancing calcium release to help with muscular contraction [175]. Of these mechanisms, it is the competitive inhibition of adenosine receptors and subsequent central nervous system stimulation that provides the most significant basis for improved exercise performance [152,153,154]. This mechanism may explain the reported suppressed feelings of discomfort and pain experienced [158,165,176] and the attenuated ratings of perceived exertion (RPE) [156,163] during exercise with pre-exercise caffeine consumption.

### 5.2. Effects of Timing

The timing of caffeine intake may also influence its ergogenic effects. Caffeine has a half-life of 4–6 h and peak serum caffeine levels typically occur 1–2 h after ingestion [177]. Therefore, many studies investigating the effects of caffeine on endurance performance administer caffeine 1 h prior to exercise to correspond with peak serum values [159,161,176]. This seems logical; however, the relative importance of serum caffeine levels to performance may depend on the degree of habituation of caffeine use. Bell and McLellan [167] gave users (>300 mg/day) and non-users (<50 mg/day) of caffeine 5 mg/kg of caffeine or a placebo one, three, and 6 h prior to cycling to exhaustion at 80% VO_2max_. Performance was enhanced to a greater degree in non-users *versus* users at all time points; however, the authors reported no differences in performance between time points for non-users despite serum caffeine levels dropping significantly at 6 h compared to 1 h. Alternatively, for caffeine users, serum caffeine levels influenced the ergogenic effects, as performance was enhanced at one and 3 h (with no between-time-point differences), which corresponded with peak serum levels, but not 6 h. These results suggest that for caffeine users, deriving an ergogenic effect from its consumption may require aligning exercise with peak serum caffeine levels (1–2 h), whereas non-users of caffeine seem to benefit from caffeine consumption regardless of its levels in the blood. However, in another study by the same authors [178], caffeine appeared to enhance performance in habituated caffeine users (>300 mg/day) in two repeated 80% VO_2max_ TTE trials separated by 5 h despite a lowering of serum caffeine levels during the between-exercise period. While it is possible that the repeated exercise altered the sensitivity to serum caffeine via downregulation of adenosine receptors [179], these results seems to contradict the findings of the previous study. Regardless, whether or not aligning exercise with peak serum caffeine levels is optimal, it remains the logical recommendation, as there is no evidence of this attenuating performance, and many studies report enhanced performance in caffeine users and non-users in this window of time.

### 5.3. Beetroot Juice (Dietary Nitrate)

Beetroot juice has garnered much attention recently for its ability to enhance endurance performance, because of its high nitrate content (for a thorough review, see [180]). Nitrate can be converted to nitric oxide in the body, which improves the vasodilation of the blood vessels, ultimately increasing blood flow to working muscles [181,182]. This attribute of dietary nitrate suggests a benefit for increased oxygen kinetics and nutrient uptake during exercise. Specifically, dietary nitrate from beetroot juice is thought to improve the efficiency of oxidative phosphorylation at a given relative intensity and reduce the breakdown of phosphocreatine (PCr) [183,184]. These mechanisms may explain the reported reductions in blood pressure and enhanced muscular efficiency during submaximal exercise with beetroot juice ingestion [184].

Pre-exercise ingestion of beetroot has been shown to enhance performance. Murphy *et al.* [185] found that 200 g of baked beetroot (≥500 mg nitrate) consumed 75 min pre-exercise improved running speed and decreased RPE during the final 1.8-km (1.1 mi) of a 5-km TT [185]. Similarly, in cyclists, Lansley and colleagues [183] reported improved TT performance with the supplementation of beetroot juice (0.5 L) in both 4-km (+2.8%) and 16-km (+2.7%) cycling TT. Multiple other recent studies confirm these findings [186,187,188]. Of interest, these benefits may be further enhanced by the addition of caffeine [189].

Worth noting, while many of the studies have seen improvements in endurance performance with beetroot supplementation, its effects might be influenced by training status, with it being most effective in low to moderately trained athletes. Christensen *et al.* [190] tested highly trained athletes with a VO_2max_ of 72 ± 4 mL/kg/min with the same amount of beetroot juice as many other studies (0.5 L) and found no improvements in performance. Similarly, Peacock *et al.* [191] tested ten male junior elite cross-country skiers (mean VO_2max_ of 74 ± 8 mL/kg/min) with potassium nitrate supplementation. Two and a half hours prior to a 5-km run on an indoor track, subjects were given either a capsule of 1 g of potassium nitrate (614 mg nitrate) or a capsule of 1 g of maltodextrin. While plasma nitrate levels were significantly increased, they found no improvements in TT with the nitrate supplementation. These results suggest that beetroot juice and/or nitrates may not enhance performance in elite endurance athletes.

## 6. Conclusions

Consuming a CHO-rich meal in the hours prior to endurance exercise appears to benefit performance. Performance may also be improved, or at least does not seem to be impaired, by ingesting CHO within 60 min of exercise. The effect of modified starches, such as Vitargo^®^ (fast-digesting) and UCAN^®^ (slow-digesting), is dependent primarily on the manner in which the starch is modified. Starches modified for the purpose of rapid digestibility can initiate rapid glycogen resynthesis, thus yielding potential performance benefits in repeated exercise. In contrast, starch modification for the purpose of slow-digestibility has been shown to increase fat oxidation during exercise compared to high-GI CHO, thus helping to preserve muscle glycogen; however, significant performance benefits have not been shown to date. High fat meals may enhance fat oxidation during subsequent exercise, although the performance effects are unclear, with most studies reporting no benefit or decrement *versus* a CHO meal. There are very few studies analyzing the performance effects of pre-exercise CHO-PRO meals, although there is some evidence suggesting potential enhanced glycogen storage and during exercise metabolism. Finally, caffeine and beetroot juice (dietary nitrates) appear to enhance performance, although these effects may be modulated by genetic factors and/or training status. More research is warranted to elucidate the effects of pre-exercise PRO, fat and modified starch consumption, as well as the effects of different pre-exercise and during-exercise nutritional combinations.
